# Alleviation of Tri-*o*-cresyl Phosphate-Induced Myelin Sheath Damage to Sciatic Nerves by Exogenous Progesterone Pretreatment

**DOI:** 10.3390/toxics14010007

**Published:** 2025-12-20

**Authors:** Pan Wang, Xiao-Hua Song, Qi Wang, Min Yang, Hai-Yang Xu, Ming-Yuan Xu, Yi-Jun Wu

**Affiliations:** Laboratory of Molecular Toxicology, State Key Laboratory of Integrated Management of Pest Insects and Rodents, Institute of Zoology, Chinese Academy of Sciences, 1-5 Beichenxilu Road, Beijing 100101, China; wangpan@cuhk.edu.cn (P.W.); monster1song@163.com (X.-H.S.); kiana508@gmail.com (Q.W.); ymzbcs_1216@163.com (M.Y.); xuhaiyang0319@sina.com (H.-Y.X.); ming_0502@163.com (M.-Y.X.)

**Keywords:** delayed neurotoxicity, ErbB2/p-Akt pathway, hen, organophosphorus, peripheral nerve

## Abstract

Some organophosphorus compounds can induce delayed neurotoxicity, which is characterized by ataxia, also known as organophosphate-induced delayed neurotoxicity (OPIDN). The underlying mechanism of axonal degeneration and demyelination in OPIDN is still poorly understood, although progress on the studies has been made. Progesterone is an important sex hormone with a neuroprotective effect, and a decrease in progesterone level was observed in the hens with OPIDN. To investigate whether exogenous progesterone offers protective effects in OPIDN and to elucidate the underlying mechanisms, we conducted an investigation with adult hens, which is the typical model animal for OPIDN research. The hens were either administrated with a single dose of the classical OPIDN inducer tri-*ortho*-cresyl phosphate (TOCP) (750 mg/kg body weight, p.o.) or were pretreated with progesterone (2 mg/kg body weight/day, i.p.) prior to TOCP exposure. The results showed that TOCP exposure induced typical OPIDN signs in hens and caused demyelinating lesions in the sciatic nerves. The pretreatment of progesterone delayed and reduced TOCP-induced gait impairment scores and restored the decreased expression of S-100β in the sciatic nerves of TOCP-exposed hens. Moreover, progesterone alleviated the TOCP-induced demyelination of the sciatic nerves. These effects were accompanied by alterations in the protein levels of the ErbB2/p-Akt signaling pathway. These findings indicate that progesterone effectively attenuates TOCP-induced delayed neurotoxicity and protects against myelin damage. This protective effect may be associated with the suppression of TOCP-induced activation of the ErbB2/p-Akt pathway, accompanied by the restoration of S-100β expression.

## 1. Introduction

Organophosphorus compounds (OPs) are used primarily as pesticides, plasticizers, plastic softeners, flame retardants, antioxidants, and hydraulic fluids [1]. Most OPs cause acute toxicity in human and animals by inhibiting acetylcholinesterase [2], while some OPs can also induce delayed neuropathy called organophosphate-induced delayed neurotoxicity (OPIDN) through inhibition and aging of neuropathy target esterase, resulting in axonal degeneration and demyelination in the central and peripheral nervous systems within 7–14 days after exposure [3]. A large-scale outbreak of OPIDN occurred in the United States during the 1930s. Drinking “Ginger Jake” contaminated with tri-o-cresyl phosphate (TOCP) caused OPIDN in an estimated 50,000 people [4]. There were also occurrences of OPIDN in Morocco, Holland, Fiji, Yugoslavia, France, South Africa, Sri Lanka, and India. These incidents were caused by the consumption of cooking oil contaminated with lubricating oil containing TOCP, which led to paralysis in thousands of individuals [5]. Despite the widespread and catastrophic outbreaks of OPIDN, and the progress made in the study of OPIDN, the neuropathological mechanism of OPIDN is still elusive, and, to date, there is still no specific treatment available for OPIDN [2]. The axonal degeneration in OPIDN resembles classical Wallerian degeneration, which is a type of atrophic degeneration that occurs in neurons distal to the site of traumatic axonal injury [6]. Clinical manifestations of OPIDN include ataxia, muscle weakness, and subsequent severe hindlimb paralysis [7]. Adult hens have been extensively used as an animal model of OPIDN [8,9] because they are sensitive to OP-induced delayed neuropathy, and their toxicological signs are similar to those seen in humans.

Progesterone is an important sex hormone that regulates different processes of reproduction such as the menstrual cycle, embryo implantation, and pregnancy [10,11]. Progesterone has been widely used to treat gynecological pathologies or reproductive complications such as recurrent miscarriage and preterm birth [12,13]. In addition, a growing number of studies have revealed that progesterone has physiological functions in non-reproductive organs, such as the immune, bone, and nervous systems [14,15,16]. Previous studies showed that progesterone has protective effects against neurological diseases such as traumatic brain injury, spinal cord injury, peripheral nerve injury, and motor neuron diseases [17,18,19,20]. Interestingly, our previous study found that TOCP exposure alters the sex hormone levels in hens. After TOCP treatment, serum progesterone concentrations remained consistently lower than controls from day 2 post-exposure until the end of the experiment, reaching roughly one-third of the control values [21,22]. Based on these findings, we hypothesized that the reduction in progesterone may contribute to the onset and progression of OPIDN, and that exogenous progesterone supplementation could alleviate the delayed neurotoxicity. In this study, we used adult hens, the typical model animal for OPIDN research, to explore whether the exogenous sex hormone progesterone could protect against OPIDN.

## 2. Materials and Methods

### 2.1. Chemicals

TOCP (purity 96%) was purchased from Acros Organics (Geel, Belgium). Progesterone was purchased from Sigma (St Louis, MO, USA). The enhanced chemiluminescent (ECL) reagent was purchased from Beyotime Biotechnology (Nantong, China). The anti-S-100β antibody (ab52642) was purchased from Abcam (Cambridge, UK), and the anti-ErbB2 antibody (sc-33684) was purchased from Santa Cruz Biotechnology (Dallas, TX, USA). Antibodies against Akt (#9272), phosphorylated Akt (p-Akt)(#9271), and anti-rabbit IgG (H + L), F(ab’)2 Fragment (Alexa Fluor^®^ 488 Conjugate) (#4412) were purchased from Cell Signaling Technology Inc. (Danvers, MA, USA). The antibodies against β-actin (CW0096M) and horseradish peroxidase-conjugated goat anti-mouse (CW0102S) and goat anti-rabbit (CW0103S) IgG were purchased from ComWin Biotech (Beijing, China).

### 2.2. Experimental Animals and Treatment

**Figure 1:**
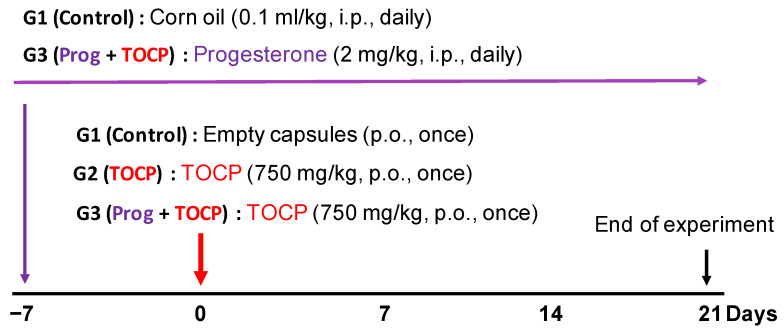
Schematic of the experimental procedure of the hens. As described in the Section 2, eighteen adult hens were randomly divided into 3 groups with six hens in each group. Empty capsules were given to the control group (group 1, G1). A single oral administration of TOCP (750 mg/kg body weight) in capsules was carried out for the other two groups (groups 2 and 3, G2 and G3). The hens in G1 received corn oil (0.1 mL/kg body weight, i.p.) daily as a vehicle for progesterone. The hens in G3 received daily administration of progesterone (2 mg/kg body weight) by intraperitoneal injection starting from the seven days before TOCP treatment until the end of the study, respectively.

Adult Beijing white laying hens aged ten months with body weight 1.3–1.5 kg were obtained from Fujia Breeding Poultry Center (Beijing, China). After one week of acclimatization, the birds were housed in an air-conditioned (22 °C) room with a 12 h light–dark cycle. Food and water were allowed ad libitum. Hens with similar body weight were randomly assigned to three groups with six hens per group. Only empty capsules were given to the control group (group 1, G1) by oral gavage. TOCP (750 mg/kg body weight) was given to all other treatment groups (groups 2 and 3, G2 and G3) by oral gavage in gelatin capsules. The dose of TOCP treatment is selected according to earlier studies [21,23,24]. The hens in group 2 (G2) received TOCP (750 mg/kg body weight, p.o.) and corn oil (0.1 mL/kg body weight, i.p.). Seven days before the TOCP treatment, the hens in group 3 (G3) received daily administration of progesterone (2 mg/kg body weight) by intraperitoneal injection until the end of the study (i.e., until the 21st day after TOCP administration); the progesterone was dissolved in corn oil. The design of the animal experiment is shown in Figure 1. The toxicological signs of OPIDN were evaluated by an eight-point scale according to a previous report [25].

### 2.3. Histopathology

Twenty-one days after TOCP exposure, the sciatic nerves of the hens were dissected and fixed in 4% paraformaldehyde overnight. The tissue samples were dehydrated through 10%, 20%, and 30% sucrose, and then embedded in O.C.T compounds for frozen sectioning. The embedded samples were then sectioned continuously at 20 μm thickness, and five sections were prepared from each sample. After permeabilization with 1% Triton X-100, the slides were blocked by using 3% BSA in the presence of 0.1% Triton X-100. Then, the sections were incubated with corresponding primary antibodies (1:50 dilution) for 1 h, followed by washing three times and incubation with secondary antibodies (1:200 dilution) for 1 h. Fluorescent staining images were taken with a Carl Zeiss LSM 710 confocal microscope (Oberkochen, Germany). The images were randomly selected for analysis and the density of S-100β-positive axons per unit area was quantified by using the ImageJ Fiji software version 1.51 (NIH, Bethesda, MD, USA).

### 2.4. Transmission Electron Microscopy

The sciatic nerve tissues were fixed in the fixative solution containing 2.5% glutaraldehyde for 24 h. The tissues were fixed in 1% OsO4, followed by dehydration through a graded ethanol series and subsequent embedding in resin. After 48 h of polymerization at 60 °C, the tissues were sliced at 200 nm or 100 nm thickness. The 200 nm slices were stained by 1% toluidine blue (dissolved in 1% Borax solution) for 1 min and observed using an Olympus IX71 optical microscope (Tokyo, Japan). The 100 nm sections were mounted onto copper grids, stained with uranyl acetate for 10 min in the dark, washed three times with PBS, and subsequently stained with lead citrate for 30 min before undergoing PBS washes. A JEOL-1010 transmission electron microscope (JEOL Ltd., Tokyo, Japan) was used to take the images.

### 2.5. Western Blotting Analysis

The sciatic nerve samples were ground with a mortar and pestle in liquid nitrogen and then sonicated in an ice-cold buffer containing 150 mM NaCl, 10 mM Tris–HCl (pH 7.5), 1% sodium deoxycholate, 0.1% SDS, 1% NP-40, 1 mM PMSF, and protease inhibitor cocktail. Tissue homogenates were centrifuged at 12,000× *g* at 4 °C for 15 min, and protein concentrations in the resulting supernatants were determined via the Bradford method [26]. Then, three samples from each group were subjected to SDS-PAGE; another three replicate samples from each group were loaded onto a second gel and run simultaneously in the same electrophoresis apparatus to ensure identical electrophoretic conditions for all samples. After electrophoresis, the proteins were transferred to PVDF membranes. The membranes were blocked with blocking solution containing 5% fat-free milk or 5% BSA for 2 h. The primary antibodies were diluted in Tris-buffered saline with Tween-20 buffer (TBST): Akt and S-100β at 1:1000, ErbB2 and p-Akt at 1:500, and β-actin at 1:2000. The membranes were incubated with primary antibodies overnight at 4 °C. After three washes with TBST, membranes were incubated with horseradish peroxidase-conjugated secondary antibody (1:2000, diluted in blocking buffer) for 2 h at room temperature. Immunoreactive bands were visualized using ECL reagents on a MicroChemi 4.2 bioimaging system (DNR, Kiryat Ono, Isreal). The band intensities for each target protein normalized to β-actin were quantified by using version 4.6.2 (BioRad, Hercules, CA, USA) and analyzed across three independent experimental replicates.

### 2.6. Statistical Analysis

SPSS Statistics 19 (IBM) was used for statistical analysis in this study. The data were shown as the mean ± SEM. Behavioral outcomes were assessed using the *Kruskal–Wallis* test, followed by Nemenyi’s post hoc comparisons, whereas other measurements were analyzed by using a one-way ANOVA followed by Dunnett’s post hoc test. Significance was set at *p* < 0.05.

## 3. Results

### 3.1. Progesterone Attenuated the Manifestations of the Delayed Neurotoxicity Induced by TOCP

**Figure 2:**
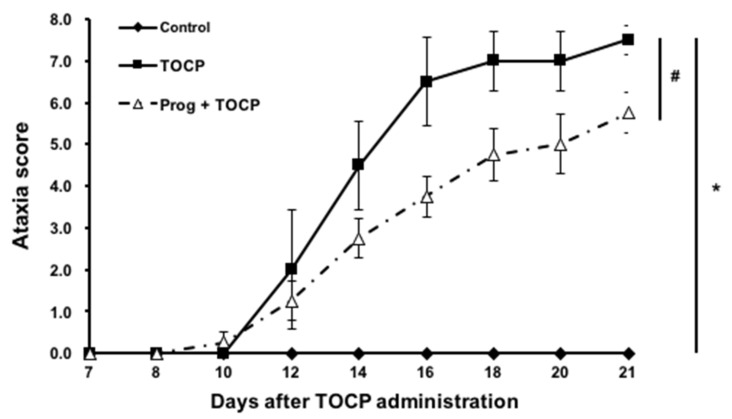
The ataxia scores of TOCP-induced delayed neurotoxicity in hens. The hens exposed to TOCP were examined daily for toxic signs on an 8-point graded scale till the end of the experiment (for details, see the Section 2). Different scores were given for severity of signs, i.e., 0 for normal ambulation; 1 and 2 for slight hindlimb incoordination; 3 and 4 for moderate hindlimb incoordination; 5 and 6 for severe difficulties in walking and standing; 7 and 8 for complete hindlimb paralysis. The ataxia score is shown as the mean ± SEM (*n* = 6). * *p* < 0.05, compared with control group; # *p* < 0.05, compared with TOCP alone treatment group. Abbreviations: TOCP, tri-*o*-cresyl phosphate; Prog, progesterone.

No hens died during the experiment. Hens exposed to TOCP alone began to exhibit ataxia on day 9 post-exposure, which became increasingly severe and eventually led to paralysis (with an ataxia score of nearly 8), while the control hens maintained a normal gait (ataxia score of 0) during the whole experimental period (Figure 2). The hens pretreated with progesterone prior to TOCP exposure exhibited a delayed onset of ataxia, which first appeared on day 12. However, the toxic signs were less pronounced in the hens treated with progesterone plus TOCP compared with those of the hens exposed to TOCP alone, and this difference persisted from the onset of ataxia until the end of the entire experiment (Figure 2). These findings suggest that pretreatment of exogenous progesterone can attenuate the delayed neurotoxicity induced by TOCP.

### 3.2. Progesterone Mitigated the TOCP-Induced Disruption of Myelin

**Figure 3:**
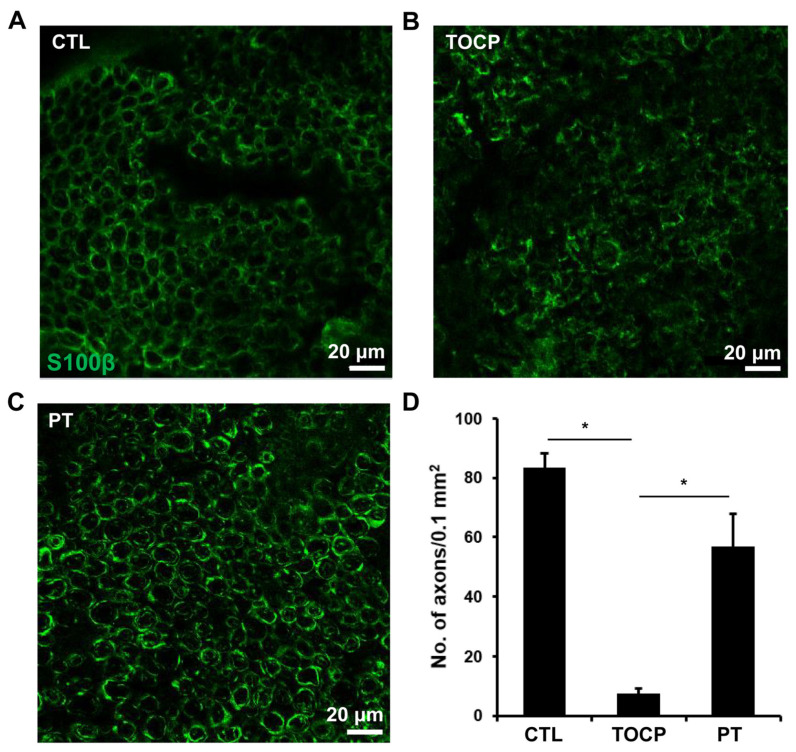
The effect of progesterone on S-100β expression in sciatic nerves of the hens exposed to TOCP. (**A**–**C**) Expression of S-100β was determined by immunofluorescence staining (green). Scale bar: 20 µm. (**D**) The quantification of S-100β expression levels in Figures (**A**–**C**). The data in Figure (**D**) are the mean ± SEM. “*” indicates significant difference between the two columns (*p* < 0.05). For quantification, one tissue block was collected from each hen in every group (six samples per group), and a mid-segment of each of the sciatic nerves (~0.5 cm) was used. Five sections were prepared from each sample, and six immunostained images per group were then randomly selected for analysis. The density of S-100β-positive axons per unit area was quantified. Abbreviations: CTL, control; TOCP, tri-*o*-cresyl phosphate; PT, progesterone plus TOCP.

**Figure 4:**
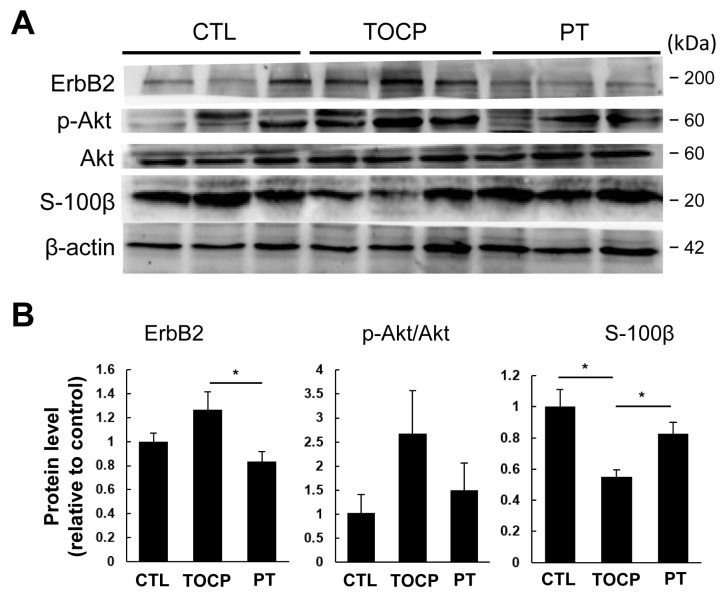
Effect of TOCP plus progesterone on expression of S-100β and ErbB2 signaling molecules in sciatic nerves of the hens. (**A**) Biochemical analysis of S-100β and ErbB signaling system by Western blotting analysis in hens’ sciatic nerves. The experiment was repeated three times. In each replicate, six samples from each group were loaded on the two gels of the same apparatus (each gel contained three samples from each group; all samples were run simultaneously). (**B**) Protein expression levels were quantified as gray values and normalized to β-actin, and the data are presented as mean ± SEM. “*” indicates significant difference between the two columns (*p* < 0.05, *n* = 3). Abbreviations: CTL, control; TOCP, tri-*o*-cresyl phosphate; PT, progesterone plus TOCP.

**Figure 5:**
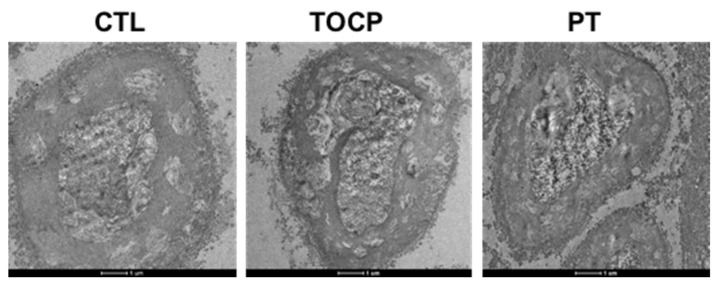
The electron microscope pictures of sciatic nerves in hens. A single dose of TOCP (750 mg/kg BW, p.o.) was given to adult hens. Seven days before the TOCP treatment, half of the hens received daily administration of progesterone (2 mg/kg BW, i.p.) until the end of the study; the other half received daily corn oil (0.1 mL/kg BW, i.p.). Control hens received only empty capsules (p.o.). For each experiment, three samples per group were randomly selected. After dissection, fixation, and embedding of the sciatic nerves, ten sections were prepared from each tissue. All sections were imaged using a transmission electron microscope under identical voltage and magnification settings. Scale bar: 1 µm. Abbreviations: BW, body weight; CTL, control; TOCP, tri-*o*-cresyl phosphate; PT, progesterone plus TOCP.

The immunostaining results on sciatic nerves showed that progesterone pretreatment partially restored the TOCP-disrupted structure of myelin sheath around the axons (Figure 3). After TOCP treatment, the protein level of S-100β, which is a marker of myelin sheath in sciatic nerves, was decreased (to approximately 50% of the control level); however, progesterone pretreatment effectively prevented TOCP-elicited S-100β loss (Figure 4). In addition, electron microscopy showed that progesterone pretreatment attenuated the axonal demyelination induced by TOCP in the sciatic nerves (Figure 5). Taken together, these findings indicate that progesterone can mitigate TOCP-induced histopathological changes and demyelination in sciatic nerves.

### 3.3. Progesterone May Exert Its Effects by Inhibiting the Activation of the ErbB2/p-Akt Signaling Pathway

It has been documented that the ErbB2/Akt signaling pathway plays an important role in regulating myelination [23,24]. It remains unclear whether progesterone protects against TOCP-induced neurotoxicity by activating the ErbB2/p-Akt pathway in the sciatic nerves. We examined the effects of progesterone on the expression of ErbB2 and its downstream molecule Akt/p-Akt in the sciatic nerves. As shown by Western blotting analysis, although the difference was not statistically significant due to large inter-sample variation, a trend toward increased ErbB2 and p-Akt levels in sciatic nerves was observed in the TOCP group compared with the control. Pretreatment with progesterone decreased the TOCP-induced elevation of ErbB2 and p-Akt levels in sciatic nerves (Figure 4). These findings indicate that progesterone attenuated the TOCP-induced S-100β loss and histopathological damage to the sciatic nerves, which may be associated with the suppression of the TOCP-activated ErbB2/p-Akt pathway.

## 4. Discussion

OPIDN is a neurodegenerative disorder that typically manifests 7–21 days or longer after OP exposure, presenting with ataxia and, in severe cases, paralysis. It is characterized by distal axonal degeneration and demyelination [3,27]. TOCP, a classic environmental contaminant that is capable of inducing OPIDN, has been detected in various environmental media and even in the human body [22,28,29]. Hens are considered an ideal animal model for studying OPIDN. In our current study, hens exposed to TOCP began to exhibit ataxia on day 9 post-exposure, and the signs gradually worsened; by the end of the experiment, most intoxicated hens were nearly paralyzed. Histopathological examination revealed pronounced demyelination in the sciatic nerves and Western blotting analysis showed that the level of S-100β, a neurotrophic factor, which is normally expressed in Schwann cells of the peripheral nervous system [30], was significantly reduced following TOCP exposure. At the molecular level, we found that TOCP activated the ErbB2/p-Akt signaling pathway, which lies upstream of S-100β regulation. These above findings suggest that demyelinating lesions in sciatic nerves occurring during TOCP-induced delayed neurotoxicity are perhaps associated with the activation of the ErbB2/p-Akt signaling pathway and the downregulation of S-100β expression.

Progesterone has been reported to exert neuroprotective effects [31,32]. In our preliminary experiments, hens treated with progesterone alone showed no differences in toxic signs or neurotoxicity indicators compared with the control hens. However, progesterone pretreatment prior to TOCP exposure alleviated neurotoxic signs and substantially reduced demyelination. Furthermore, progesterone pretreatment effectively attenuated TOCP-induced activation of the ErbB2/p-Akt signaling pathway and, to some extent, restored the expression of S-100β. These findings indicate that the protective effect of progesterone against TOCP-induced delayed neurotoxicity may involve both inhibition of the ErbB2/p-Akt signaling pathway and upregulation of S-100β expression.

The precise mechanisms by which progesterone suppresses the ErbB2/p-Akt pathway and mitigates OPIDN remain to be fully elucidated. It has been reported that neuroprotective steroid hormones including progesterone can exert their effects through multiple receptor-mediated pathways, such as the intracellular progesterone receptor (PR), membrane progesterone receptor (mPR), and γ-aminobutyric acid type A receptor (GABAAR) [33,34]. Progesterone has been shown to bind intracellular receptors and activate key myelin-related transcription factors in Schwann cells, thereby promoting the expression of myelin structural proteins. Our previous work demonstrated that TOCP significantly decreases the expression of myelin-related proteins including MBP and P0 [13]. In this study, we found that progesterone effectively alleviated TOCP-induced delayed neurotoxicity. This protective effect may result from progesterone binding to PR, mPR, or other receptors and initiating downstream neuroprotective signaling cascades. However, our experiment found that when the hens were co-administrated with mifepristone, a PR antagonist, the protective effect of progesterone on TOCP-induced delayed neurotoxicity remained unchanged (unpublished data), which suggested that progesterone may alleviate the TOCP-induced myelin damage in sciatic nerves independently of PR or mPR. It is also possible that exogenous progesterone pretreatment reduces TOCP neurotoxicity by preventing TOCP from interacting with GABAAR or other relevant receptors. Furthermore, progesterone metabolites such as allopregnenolone and dihydroprogesterone have also been reported to play important roles in peripheral nerve protection [20,35]. Thus, it is possible that the protective effects observed following progesterone administration may partially result from its metabolites. In addition, our previous study found that TOCP can provoke oxidative stress while inducing neurotoxicity [36]. Given the established antioxidant properties of progesterone and its metabolites [36,37], we cannot exclude the possibility that the attenuation of oxidative stress contributes, at least in part, to the protective effects of progesterone against TOCP-induced neurotoxicity.

Due to the lack of data on whether and how TOCP exposure affects allopregnenolone and dihydroprogesterone levels in neural tissues, our findings are insufficient to determine whether the observed protection against TOCP-induced delayed neurotoxicity is mediated primarily by progesterone itself or by its metabolites. Additionally, this study has other limitations, such as the small number of samples used for Western blotting analysis, the lack of data on MBP/P0 expression in myelin sheaths and electrophysiological examination of the sciatic nerves, and the absence of a progesterone-only treatment group. Although further experiments are needed to clarify the issues, the present work highlights promising avenues for future research aimed at elucidating the mechanisms underlying progesterone-mediated protection in OPIDN.

## 5. Conclusions

In summary, our findings demonstrate that progesterone effectively alleviates TOCP-induced delayed neurotoxicity, and this protective effect may be associated with its ability to suppress TOCP-activated ErbB2/p-Akt signaling, accompanied by the restoration of S-100β expression.

## Data Availability

All data generated or analyzed in the current study are available from the corresponding author on reasonable request. All data generated or analyzed during this study are included in this published article.
